# Independent Walking at Discharge in Nonsurgical Patients With Thalamic Hemorrhage Who Were Nonambulatory on Day 14: A Retrospective Study of Prognostic Factors

**DOI:** 10.7759/cureus.107072

**Published:** 2026-04-15

**Authors:** Ayaka Fujita, Joji Hagii, Eiichi Tsuda

**Affiliations:** 1 Department of Rehabilitation Medicine, Hirosaki University Hospital, Hirosaki, JPN; 2 Department of Internal Medicine, Hirosaki Stroke and Rehabilitation Center, Hirosaki, JPN; 3 Department of Rehabilitation Medicine, Hirosaki University Graduate School of Medicine, Hirosaki, JPN

**Keywords:** functional independence measure, prognostic factors, rehabilitation, thalamic hemorrhage, walking independence

## Abstract

Background

Thalamic hemorrhage may cause severe disability because it can affect motor, sensory, cognitive, and postural functions. However, it is often difficult to estimate later walking outcomes during early hospitalization, especially in patients who are still non-ambulatory 14 days after disease onset.

Objective

To identify the factors associated with independent walking at discharge in non-surgical patients with thalamic hemorrhage who were non-ambulatory on day 14.

Methods

We conducted a retrospective cohort study at a single stroke rehabilitation center and reviewed consecutive patients with acute thalamic hemorrhage admitted between April 2016 and March 2021. The initially eligible cohort comprised 101 patients. In this revised analysis, 19 patients who had already regained independent walking by day 14 were excluded, leaving 82 patients for the final analysis. The primary outcome was independent walking at discharge, defined as a Functional Independence Measure (FIM) walk item score of 6 or 7. Clinical variables were collected from the medical records, and a multivariable logistic regression analysis was performed.

Results

Of the 82 patients, 39 achieved independent walking at discharge. The independent walking group showed younger age, lower National Institutes of Health Stroke Scale scores at admission, and higher day-14 FIM motor scores than the non-independent walking group. The multivariable logistic regression model showed that younger age (odds ratio [OR], 0.88; 95% confidence interval [CI], 0.80-0.95; p = 0.0003) and a higher FIM motor score on day 14 (OR, 1.21; 95% CI, 1.12-1.33; p < 0.0001) were independently associated with walking independence at discharge.

Conclusions

In non-surgical patients with thalamic hemorrhage who were non-ambulatory on day 14, younger age and better motor function on day 14 were associated with independent walking at discharge. In our setting, a simple bedside functional assessment on day 14 may help estimate walking status at hospital discharge. However, these findings should be interpreted cautiously, as discharge walking status may also be influenced by rehabilitation practice and institutional factors.

## Introduction

Intracerebral hemorrhage (ICH) is a serious cause of mortality and long-term disability [1,2]. Thalamic hemorrhage is a clinically important subtype because the thalamus contributes to arousal, attention, and cognition [3]; therefore, thalamic hemorrhage may cause broad neurological deficits [4]. Accordingly, patients with thalamic hemorrhage may present with a broad range of impairments. These include motor weakness, sensory deficits, disturbed consciousness, and impaired postural control [4,5], as well as attentional and memory problems related to thalamic dysfunction [3,4].

Many previous studies have examined mortality and global functional outcomes after thalamic hemorrhage [4,5]. Some studies have reported factors associated with walking recovery after stroke, such as age, sitting balance, and early functional status [6,7]. However, few studies have specifically examined walking independence during hospitalization in patients with thalamic hemorrhage who remain non-ambulatory at two weeks after onset. Previous research has addressed walking recovery after thalamic hemorrhage in a rehabilitation setting [5]. However, little is known about patients who remain non-ambulatory early after onset.

This subgroup is clinically important because prognostic uncertainty is often greatest at this stage, when rehabilitation planning and discharge preparation must proceed despite persistent walking disability. This study aimed to identify the factors associated with independent walking at discharge in non-surgical patients with thalamic hemorrhage who were non-ambulatory on day 14.

## Materials and methods

Patients and study design

We performed a retrospective cohort study at a single stroke rehabilitation center in Japan that provides both acute stroke care and continued in-hospital rehabilitation. We reviewed consecutive patients with acute thalamic hemorrhage who were admitted between April 2016 and March 2021. Thalamic hemorrhage was diagnosed by stroke specialists based on clinical findings and computed tomography. Rehabilitation was initiated within 24 hours of admission, unless the patient’s general condition precluded it. In our institution, the standard schedule generally consisted of 40 minutes each of physical therapy and occupational therapy on five days per week, with 20 minutes of either therapy on the remaining two days. However, rehabilitation intensity and content were adjusted according to the patient’s condition and institutional circumstances. In our institution, patients with stroke typically receive acute-phase treatment during the first 14 days after onset and are then transferred to a convalescent rehabilitation ward for rehabilitation-centered care. For this reason, day 14 represents a clinically meaningful time point for functional assessment and discharge planning in our setting.

**Figure 1:**
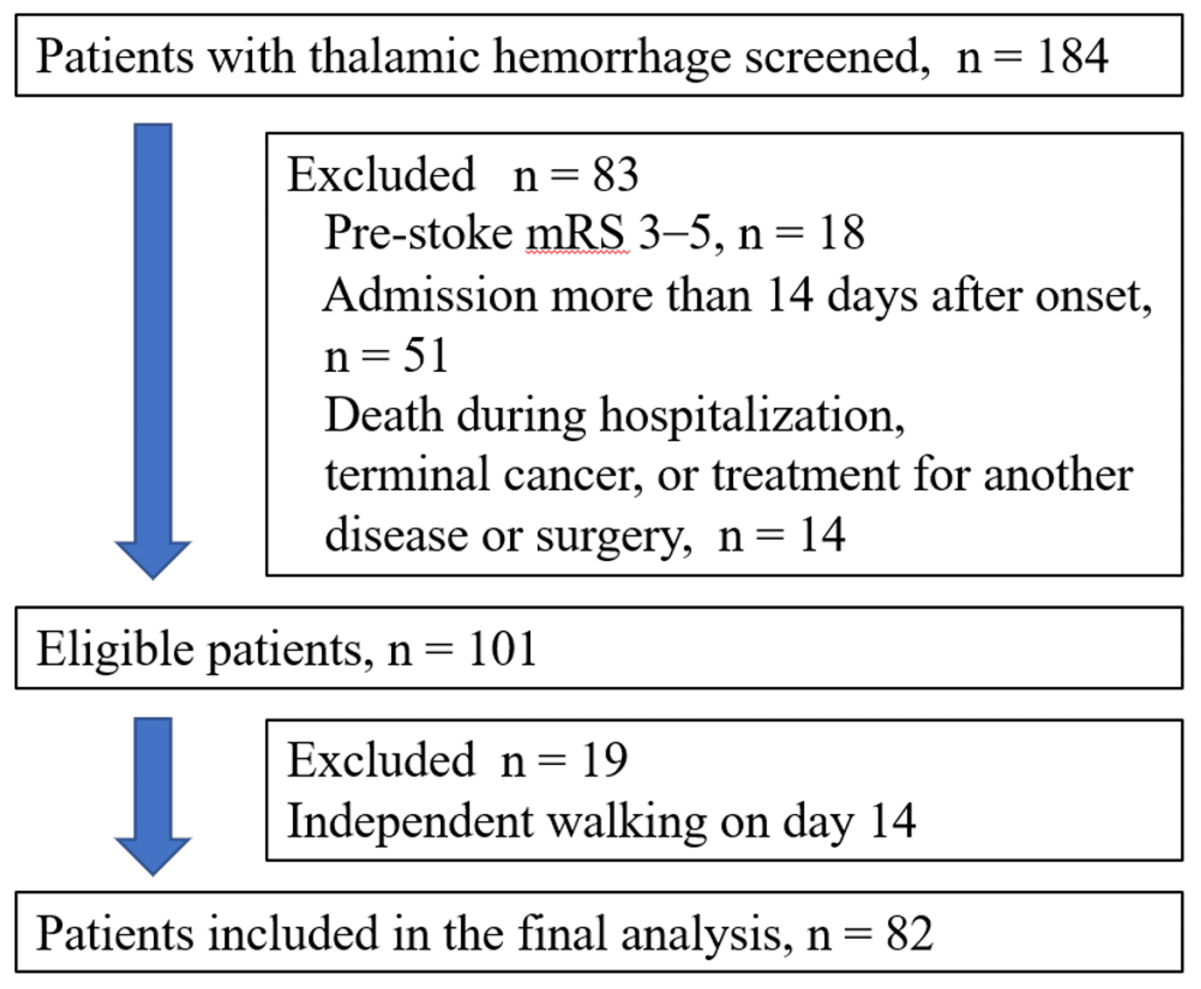
Flowchart of patient inclusion and exclusion. A total of 184 patients with thalamic hemorrhage were screened during the study period. After application of the exclusion criteria, 101 patients were eligible for the study. Nineteen patients who had already achieved independent walking on day 14 were further excluded. Finally, 82 patients were included in the analysis. mRS: modified Rankin Scale.

The original eligible cohort included 101 patients. Patients were excluded if they were admitted > 14 days after disease onset, had pre-stroke dependence in activities of daily living, underwent surgery, required treatment for another severe acute disease, had terminal cancer, or died during hospitalization. In the present revised analysis, we also excluded 19 patients who had already achieved independent walking on day 14 because the aim of this study was to identify prognostic factors among patients who remained non-ambulatory at that time point. Including patients who had already regained walking ability would have introduced a different clinical population and potentially obscured factors relevant to delayed recovery. Therefore, a total of 82 patients were analyzed (Figure 1).

Ethical approval

The study protocol was reviewed and approved by the Ethics Committee of the Hirosaki Stroke and Rehabilitation Center (protocol number 21A008) and was conducted in accordance with the Declaration of Helsinki. Because of the retrospective study design, the ethics committee waived the requirement for written informed consent. Patients were allowed to opt out through the hospital website.

Outcome

The primary outcome was independent walking at discharge from our hospital. Walking ability was assessed using the walk item of the Functional Independence Measure (FIM) [8]. Independent walking was defined as an FIM walk score of 6 or 7.

Clinical variables

The following variables were collected from the medical records: sex, age at onset, days from onset to admission, National Institutes of Health Stroke Scale (NIHSS) score [9] on admission, pre-stroke modified Rankin Scale (mRS) score [10], right-sided thalamic hemorrhage, thalamic hemorrhage type, hematoma volume, intraventricular hemorrhage, history of stroke, FIM motor score on day 14, FIM cognitive score on day 14, sitting independence on day 14, and walking status at discharge.

Classification of thalamic hemorrhage

**Figure 2:**
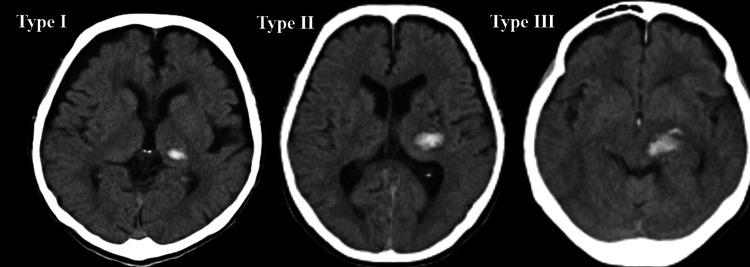
Classification of thalamic hemorrhage on computed tomography. Representative axial non-contrast computed tomography images of thalamic hemorrhage are shown. Type I was confined to the thalamus. Type II extended into the internal capsule. Type III reached the midbrain.

Thalamic hemorrhage was classified into three types according to the extent of hematoma on computed tomography: Type I, confined to the thalamus; Type II, extending into the internal capsule; and Type III, reaching the midbrain (Figure 2).

Hematoma volume

The hematoma volume was calculated using the ABC/2 method [11].

Statistical analysis

Continuous variables are summarized as median (interquartile range), and categorical variables are expressed as number (%). Between-group differences were assessed with the Mann-Whitney U test for continuous or ordinal variables and with the chi-square test or Fisher's exact test for categorical variables, depending on the data structure. Absolute Z values are presented for Mann-Whitney U tests. All statistical tests were two-tailed, with P < 0.05 considered statistically significant. Statistical analyses were conducted using JMP Student Edition version 18.2.0 (SAS Institute Inc., Cary, NC, USA).

We used multivariable logistic regression analysis to examine independent predictors of walking independence at discharge. Although hematoma volume and intraventricular hemorrhage were significant in univariable analyses, they were not included in the final multivariable model because the number of outcome events was limited and inclusion of additional variables could increase the risk of overfitting [12]. Given the limited number of outcome events, we prioritized variables that were clinically interpretable and directly relevant to functional recovery. In addition, hematoma volume and intraventricular hemorrhage may partly reflect overall disease severity, which may also be captured by the FIM motor score on day 14. Candidate variables were reviewed based on clinical relevance, strength of association in univariable comparisons, and potential collinearity. Age and the FIM motor score on day 14 were selected for the final model because they were clinically interpretable, strongly associated with the outcome, and represented complementary domains of prognosis, namely baseline patient factor and early functional status. Sitting independence on day 14 was not entered into the final model because it was considered to overlap conceptually with early functional status, as represented by the FIM motor score. There were no missing values in the variables included in the final analysis. Reporting of this observational study followed the Strengthening the Reporting of Observational Studies in Epidemiology (STROBE) recommendations.

## Results

Study population

The original eligible cohort included 101 patients with thalamic hemorrhage. In this revised analysis, 19 patients who had already achieved independent walking on day 14 were excluded. Therefore, the final analysis included 82 patients (Figure 1).

Baseline characteristics

Of the 82 patients who were non-ambulatory on day 14, 39 achieved independent walking at discharge and 43 did not (Table 1).

**Table 1:** Baseline and day 14 clinical characteristics according to walking outcome at discharge Data are presented as n (%) or median (interquartile range). mRS: modified Rankin scale, NIHSS: National Institutes of Health Stroke Scale, FIM: Functional Independence Measure

Variables	Independent walking (n = 39)	Non-independent walking (n = 43)	Test statistic	p-value
Age, years	68 (59–78)	77 (70–85)	Z = 3.42	p = 0.0006
Male sex, n (%)	25 (64.1)	19 (44.2)	χ² = 3.29	p = 0.070
Pre-stroke mRS, n (%)	N/A	N/A	Fisher’s exact test	p = 0.0552
mRS 0, n (%)	33 (84.6)	26 (60.5)	N/A	N/A
mRS 1, n (%)	4 (10.3)	10 (23.3)	N/A	N/A
mRS 2, n (%)	2 (5.1)	7 (16.3)	N/A	N/A
History of stroke, n (%)	25 (64.1)	26 (60.5)	χ² = 0.115	p = 0.734
Right-sided thalamic hemorrhage, n (%)	18 (46.2)	22 (51.2)	χ² = 0.205	p = 0.650
Hematoma volume (mL)	4.5 (2.0–9.6)	7.4 (4.2–10.6)	Z = 2.40	p = 0.017
Intraventricular hemorrhage, n (%)	17 (43.6)	29 (67.3)	χ² = 4.724	p = 0.030
Thalamic hemorrhage classification, n (%)	N/A	N/A	Fisher’s exact test	p < 0.0001
Type I, n (%)	19 (48.7)	4 (9.3)	N/A	N/A
Type II, n (%)	20 (51.3)	33 (76.7)	N/A	N/A
Type III, n (%)	0 (0)	6 (14.0)	N/A	N/A
Days from onset to admission	0 (0–0)	0 (0–1)	Z = 0.52	p = 0.600
NIHSS score on admission	9 (5–13)	15 (12–21)	Z = 4.36	p < 0.0001
Sitting independence on day 14, n (%)	19 (48.2)	1 (2.3)	Fisher’s exact test	p < 0.0001
FIM motor score on day 14	40 (25–52)	13 (13–19)	Z = 6.46	p < 0.0001
FIM cognitive score on day 14	20 (14–27)	9 (5–13)	Z = 5.24	p < 0.0001

The independent walking group was younger than the non-independent walking group. They also had lower pre-stroke disability, smaller hematoma volumes, less frequent intraventricular hemorrhage, less severe hemorrhage classification, lower NIHSS scores on admission, and higher FIM motor and cognitive scores on day 14. Sitting independence on day 14 was also more common in the independent walking group.

Multivariable logistic regression

Age and the FIM motor score on day 14 remained independently associated with walking independence at discharge in the multivariable model. Older age was independently associated with lower odds of independent walking (adjusted OR, 0.88; 95% CI, 0.80-0.95; p = 0.0003), whereas a higher FIM motor score on day 14 was independently associated with greater odds of independent walking (adjusted OR, 1.21; 95% CI, 1.12-1.33; p < 0.0001) (Table 2).

**Table 2:** Multivariable logistic regression analysis for independent walking at discharge FIM: Functional Independence Measure

Variables	Odds ratio	95% Confidence Interval	P-Value
Age (years)	0.88	0.80-0.95	P=0.0003*
FIM motor score on day 14	1.21	1.12-1.33	P<0.0001*

## Discussion

This study focused on non-surgical patients with thalamic hemorrhage who remained unable to walk 14 days after disease onset. In this subgroup, younger age and a higher FIM motor score on day 14 were independently associated with independent walking at discharge. In our setting, a bedside assessment at two weeks may provide useful information for estimating walking status at hospital discharge.

Previous studies in stroke populations have reported that age, sitting balance, lower limb function, and early functional performance are related to subsequent walking recovery [13,14]. In contrast, previous reports on thalamic hemorrhage have largely emphasized mortality or broader functional outcomes rather than in-hospital walking independence [4,5]. The present study addressed a more specific clinical problem by examining patients who were still non-ambulatory on day 14. This is the stage at which rehabilitation teams must make decisions about prognosis, treatment planning, and discharge preparation.

In our cohort, the FIM motor score on day 14 was the strongest predictor of walking independence at discharge. This result is clinically plausible because the motor FIM score captures several elements of early recovery, including transfers, trunk control, and basic self-care. In patients with thalamic hemorrhage, a low motor FIM score may indicate not only motor weakness but also reduced attention, impaired arousal, and more extensive early disability. Therefore, the motor FIM score may be better understood as a practical summary of overall early functional severity rather than as an isolated measure of limb motor impairment. This interpretation is in line with previous work showing that early functional motor performance strongly influences discharge outcomes after stroke [14]. In addition, the strong association between the day-14 FIM motor score and independent walking at discharge should be interpreted cautiously. Although the FIM motor score represents overall early functional status, it also includes abilities that are closely related to walking. Therefore, some conceptual overlap with the outcome cannot be excluded.

Age was independently associated with walking outcomes. This finding is consistent with previous stroke studies, which have shown that younger patients are more likely to regain independent walking [13, 14]. Age is not modifiable; however, it remains an important background factor when clinicians discuss prognosis with patients and families during the early phase of hospitalization.

Sitting independence on day 14 was also clinically informative. Sitting balance and trunk control are known to be related to later walking recovery after stroke [13, 15]. In our cohort, sitting independence was more common in patients who later achieved independent walking. This finding supports the value of simple bedside assessments during early rehabilitation. However, sitting independence was not included in the final regression model because we aimed to keep the final model simple and avoid overfitting, and because it conceptually overlapped with early functional status represented by the FIM motor score.

Several important contextual factors should be considered when interpreting our findings. First, the outcome of independent walking at discharge is not solely determined by neurological recovery. It may also be influenced by institutional factors, including rehabilitation intensity, timing of mobilization, length of hospital stay, and discharge policies. Therefore, the observed associations between early functional status and walking independence should be interpreted within the specific clinical context of our center rather than as universally applicable predictors.

Second, the use of day 14 as a prognostic time point requires careful consideration. Although this time point is clinically practical in our setting, as it often corresponds to a stage when rehabilitation planning and discharge decisions are actively discussed, it may not represent a universally optimal or biologically distinct phase of recovery. The trajectory of functional recovery after stroke can vary across patients and healthcare systems, and alternative time points may be equally or more informative in different contexts.

Taken together, these considerations suggest that our findings provide context-specific insights rather than definitive prognostic rules. Further studies in diverse settings are needed to determine the generalizability of these results and to clarify the role of early functional assessments at different time points.

This study had several strengths. First, it focused on a clinically important subgroup of patients with thalamic hemorrhage who remained non-ambulatory at two weeks. Second, it used simple bedside measures that are easy to obtain in routine practice.

Third, the study design directly addressed the methodological problem of including patients who had regained independent walking on day 14 in the prognostic analysis.

The present results should be interpreted with caution because of several limitations. In particular, this was a retrospective study performed at a single center, and selection bias cannot be ruled out. Second, the sample size was limited, and external validation was not performed. Third, although inpatient rehabilitation was provided based on the general practice of our institution, the actual intensity and content of therapy varied according to each patient's condition and institutional circumstances. Therefore, the potential influence of rehabilitation variability on walking outcomes could not be fully evaluated. Fourth, patients who underwent surgery or had very severe disease were excluded; therefore, these findings may not apply to all patients with thalamic hemorrhage. Fifth, discharge walking status may have been influenced not only by functional recovery but also by institutional length of stay and discharge policies. Sixth, the use of day 14 as a prognostic time point reflects the clinical workflow of our institution and may not be directly applicable to other settings, where prognostic assessment may be performed at different stages of hospitalization. Finally, although hematoma-related variables such as hematoma volume and intraventricular hemorrhage were associated with walking outcomes in univariable analyses, they were not included in the final multivariable model due to the limited number of outcome events and the need to avoid overfitting. As a result, the observed associations, particularly for the FIM motor score, may partly reflect underlying disease severity.

## Conclusions

Among nonsurgical patients with thalamic hemorrhage who were nonambulatory on day 14, younger age and better motor function on day 14 were associated with independent walking at discharge. In our setting, assessment of early motor function may help estimate walking status by the time of hospital discharge and may support rehabilitation planning and discussions with patients and families. However, these findings should be interpreted cautiously because discharge walking status may also be influenced by rehabilitation practices, length of stay, and institutional discharge policies. Further prospective multicenter studies are needed to confirm the generalizability of these findings.
